# Improvement in Early Scar Maturation by Nanofat Infiltration: Histological and Spectrophotometric Preliminary Results From a Split Scar–Controlled, Randomized, Double-Blinded Clinical Trial

**DOI:** 10.1093/asjof/ojae072

**Published:** 2024-08-30

**Authors:** Lisa Ramaut, Linde Moonen, Maxim Geeroms, Gaelle Leemans, Ellen Peters, Ramses Forsyth, Jan Gutermuth, Moustapha Hamdi

## Abstract

**Background:**

The regenerative properties of stromal vascular fraction (SVF) in wound healing and scar formation are a subject of increasing clinical interest.

**Objectives:**

Although preclinical studies have confirmed the angiogenetic, proliferative, and antifibrotic properties of SVF, there is limited clinical evidence from randomized controlled clinical trials.

**Methods:**

Twelve patients who underwent abdominoplasty were included in this clinical study. Nanofat was mechanically obtained intraoperatively and infiltrated intradermally in the sutured surgical wound, randomly assigned to either the left or the right side. The abdominal scar was evaluated with the Patient and Observer Scar Assessment Scale, whereas erythema and pigmentation were measured with a reflectance spectrophotometry device (Mexameter, Courage + Khazaka electronic GmbH, Köln,Germany). Histological analysis and electron scan microscopy of tissue biopsies were performed at 8 months.

**Results:**

The treated side of the scar showed significantly less erythema at 3- and 6-month follow-ups, but this difference reduced after 12 months. Patients reported better scar scores at the 6-month follow-up with a significantly better color at the treated side. Observers reported better overall scar scores at the treated side at 3-, 6-, and 12-month follow-ups, with better vascularization, pigmentation, and thickness. There was no statistically significant difference in terms of histological analysis between the 2 groups. There was no difference in the occurrence of adverse events between both sides.

**Conclusions:**

Infiltration of nanofat exhibited promising results in surgical scar maturation characterized by less erythema and better texture. More clinical trials with a larger sample size are warranted to better elucidate the possible benefits of SVF on surgical scar formation.

**Level of Evidence: 5:**

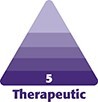

The benefits of fat grafting on skin quality and scar tissue is a well-known phenomenon. Experts agree that these effects can be attributed to the stromal component of adipose tissue rather than to the adipocyte-containing parenchyma. From this perspective, the promising field of fat grafting in scar treatment shifted to the use of a more concentrated substrate that is devoid of adipocytes, called stromal vascular fraction (SVF).^1-5^ SVF resides in all tissues and is responsible for tissue regeneration and repair through their specialized differentiation potential of stem and progenitor cells and release of cytokines and growth factors.^6–10^ It was Tonnard and Verpaele who introduced a bedside SVF therapy from easily accessible lipoaspirate and named it Nanofat.^9^ In vitro research revealed that adipose-derived SVF has specific immunomodulatory, angiogenetic, and antifibrotic properties, which are interesting assets in the search for scar therapies that aim to mimic scarless wound healing as seen in fetal wound suturing.^11^ A shortened inflammatory phase and almost absent fibrotic response are characteristics of this phenomenon seen in utero.^12^ In this regard, a prolonged inflammatory phase will impair dermal regeneration and trigger the surrounding tissues in an attempt to restore the breach in the epidermal barrier by the lay down of excessive scar tissue that is less stable and more prone to hypertrophy and contractures.^13^ Balanced scar tissue formation with less fibrosis, less activated myofibroblasts, and diminished extra-cellular matrix (ECM) production is seen when the inflammation in scar tissue is tempered.^12,14,15^ In vitro research revealed the immunomodulatory and antifibrotic properties of SVF by means of reduced collagen deposition and increased scar tissue remodeling, phenomena that are impaired in pathological scarring and hypertrophy. The suppression by SVF of inflammatory cytokines and induction of apoptosis in activated macrophages play a role in the inhibition of the fibro-inflammatory process.^14,16-19^ This prolonged inflammation that leads to pathological scar formation is clinically visible by prolonged erythema from increased neoangiogenesis and persisting vascularity.^13^

The purpose of this study is to evaluate whether the infiltration of nanofat in a fresh wound influences scar maturation and quality. Surgical scars are still an inevitable by-product of surgical access to the human body. The search for modalities that protect the surgical patient from the formation of excessive scar aspires to improve surgical outcomes and speed up recovery from surgery. Nevertheless, surgical scars are intentional and reproducible, making them a suitable study target in scar treatment studies. The abdominal scar after abdominoplasty was chosen to be the target of a randomized, within-patient controlled, double-blinded clinical trial that evaluates whether intraoperative infiltration of nanofat in surgical wounds influences scar maturation and the final long-term scar quality and appearance.

## METHODS

### Study Sample

Between July 2019 and June 2021, patients who underwent abdominoplasty were recruited at our department. The surgical wound of the abdomen was included in this study to be infiltrated intradermally covering half of its length; the side was chosen randomly. The exclusion criteria were as follows: smokers, patients with connective tissue disease, patients under immunosuppressive medication, patients with diabetics, patients younger than 18 years or older than 65 years, and patients with a BMI below 18 or above 30. This study was in concordance with the Declaration of Helsinki and approved by the institutional ethics committee and was registered on clinicaltrials.gov under ID NCT03850119. All patients signed the written informed consent form prior to their inclusion. The study was put on hold during the COVID pandemic and, therefore, took longer than expected to finish.

### Preparation of the Adipose-Derived Stromal Vascular Fraction

During the surgical intervention, adipose tissue was aspirated after infiltration with a saline/adrenaline solution from the upper abdomen and flanks. A manual negative pressure liposuction was performed using a 3 mm blunt cannula with sharp side holes attached to a 10 cc syringe. The lipoaspirate was centrifuged for 1 min at 1200 rpm to separate and discard the watery portion. The remaining adipose tissue was emulsified by pushing it back and forth between 2 Luer-Lock 10 cc syringes interconnected by a Tulip Nanofat Transfer connector (Tulip Medical, San Diego, CA; 30 times through the 2.4 mm–diameter connecting piece, 30 times through the 1.2 mm–diameter connecting piece). The emulsified fat was filtered over a 600 to 400 µm metal grid filter to remove fibrous remnants that clog up 27G needles.

### Treatment Protocol

The abdomen was closed in a 3-layered fashion with intradermal sutures in the skin. The entire length of the donor site was closed by the same surgeon to eliminate bias of different suture methods in each half of the scar. The surgical wound was divided into 2 equal halves, and each half was randomized in a treatment arm (intradermal injection of SVF or no injection of SVF). The untreated side served as within-patient control. When the abdominal wound was sutured, 5 cc of nanofat was injected in the wound edges over the entire length of the scar with blanching of the skin as the endpoint. Skin glue and a dry aseptic bandage were applied over the entire wound. Postoperatively, patients were instructed to wear a supportive abdominal band for 6 weeks. The dry aseptic bandage was replaced every 3 days until the wound was closed. Notably, both patients and assessor remained blinded to the treatment protocol throughout the assessment/study period.

### Demographic Data and Outcome Measurement

Demographic data and patient characteristics were obtained from patients’ electronic files. Patients were seen at the outpatient clinic at 1, 2, and 4 weeks and at 3, 6, and 12 months. Both patient- and observer-reported clinical outcomes were quantified by using the Patient and Observer Scar Assessment Scale (POSAS) questionnaire, whereas scar erythema and architecture were objectified by performing spectrophotometric and histological analyses. Because patients with wounds and scars are a very heterogenic population, it is a challenge to eliminate confounding factors when treatment protocols are being investigated. In an etiology that is influenced immensely by patient-related confounding factors, the most optimal study design is one with within-patient control. This allows the observer to differentiate between the effect of the studied treatment and the natural course that is heavily influenced by the patients’ individual characteristics. The 2 sides of the scar were evaluated by means of the POSAS version 2.0 (POSAS, Beverwijk, The Netherlands). The observer score was filled in by a plastic surgeon blinded for the treatment. The surface section was not filled in because the scar has a linear shape. Photographs were taken and adverse events were registered during each follow-up visit.

### Histological Assessment

Tissue biopsies of the scar were obtained at 8 months from each side of the scar, halfway between the lateral edge and the midline. They were formalin-fixed and embedded in paraffin for section. They were deparaffinized and stained with hematoxylin and eosin (H&E) to evaluate epidermal and dermal architectures. A Fontana stain was used to evaluate the amount of pigmentation at the basal layer. Picrosirius red staining and Collagen I (EPR 7765 Abcam Limited, Cambridge, United Kingdom) and Collagen VI (EPR 17077, Abcam) immunohistochemistry were used to evaluate collagen organization. They were analyzed by light microscopy. Samples were fixed in glutaraldehyde and sectioned for electron microscopy (MegaView G2 CCD camera; SIS Company, Munster, Germany).

### Spectrophotometry

Spectrophotometry measures the amount of hemoglobin (reflecting 660 nm wavelengths in the red oxygenated state) and deoxyhemoglobin (reflecting 940 nm wavelengths) in the dermis and translates this to an index score of erythema.^20^ At 3-, 6-month, and 1-year follow-up visits, the erythema and pigmentation index of the abdominal scar was measured using a narrow-band skin reflectance spectrophotometer (Mexameter, Courage + Khazaka electronic GmbH, Köln, Germany). Each side of the scar was measured at 4 points, and the mean of all points of each side was compared with the mean value on the other side.

### Statistical Analysis

A statistical analysis of the gathered data was performed with Prism 8 software (GraphPad, La Jolla, CA). For both paired and unpaired data from the POSAS questionnaires, histological and spectrophotometric analyses were performed using a *t* test when data were confirmed by the Shapiro–Wilk normality test. A 2-sided *P* < .05 was considered statistically significant.

## RESULTS

### Demographic Data

A total of 15 patients were included in this study. Two patients were lost to follow-up before the 6-month visit and therefore excluded. One patient had revisional surgery for a persistent seroma and was therefore also excluded from the analysis. The mean age was 43.9 years (range, 31-61 years), and the mean BMI was 25.1 (range, 22.3-29.9). All patients were female; none of them were smokers or suffered from diabetes (Table 1).

**Table 1.** Demographic Data

Age	
18-30	0
31-40	6
41-60	5
>50	2
Mean	43.9
Range	31-61
BMI	
18-25	8
25-30	5
Mean	25.1
Range	22.3-29.9
Sex	
Male	0
Female	13
Smoking	
Yes	0
No	13
Diabetes	
Yes	0
No	13

#### Nanofat Infiltration in the Surgical Wound Improves Early Clinical Scar Maturation Reported by Patients and Observers But Does Not Significantly Improve the Final Scar Appearance

**Figure 1.**
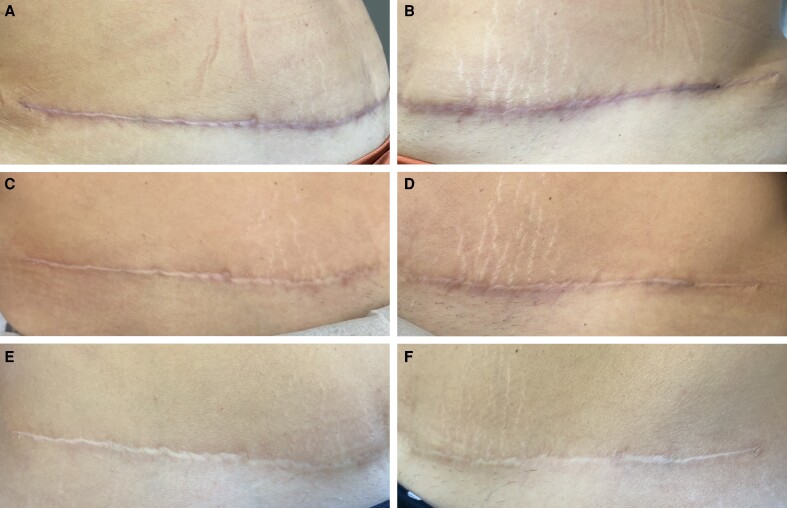
Clinical images of the abdominal scar of a 39-year-old female study patient: (A) the treated side at the 1-month follow-up; (B) the control side at the 1-month follow-up; (C) the treated side at the 6-month follow-up; (D) the control side at the 6-month follow-up; (E) the treated side at the 1-year follow-up; (F) the control side at the 1-year follow-up.

**Figure 2.**
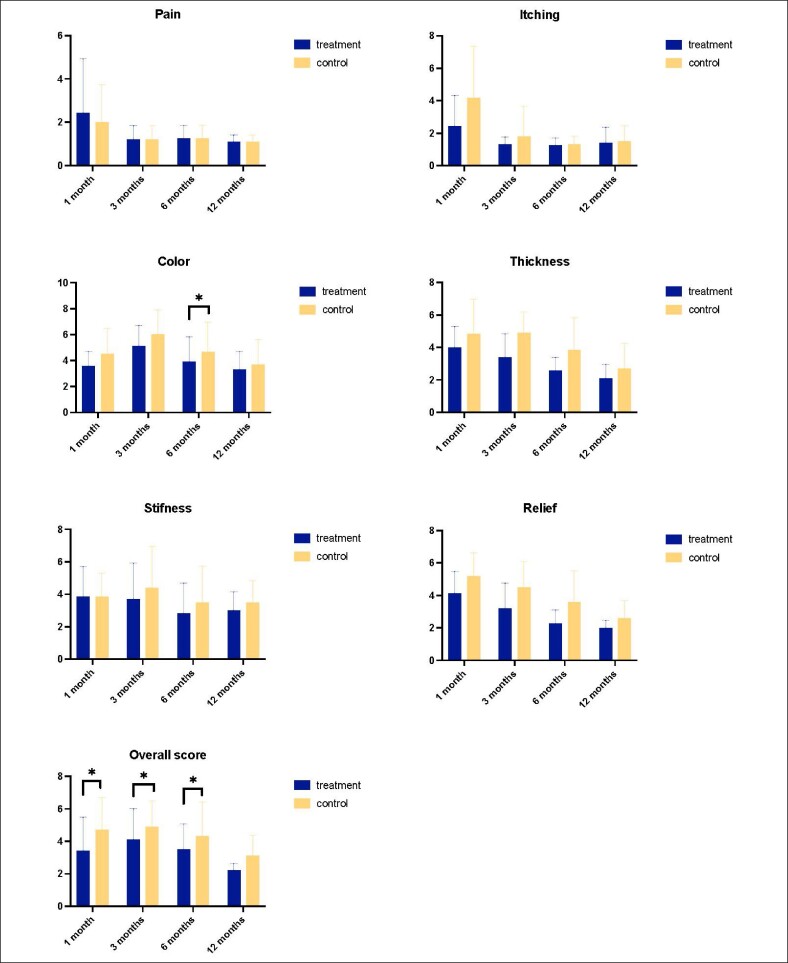
A graphical representation of patient-reported outcomes measured by using the Patient and Observer Scar Assessment Scale questionnaire (*P < .05).

On the POSAS questionnaire, patients reported a significantly better texture of the scar at the treated side at the 1-month follow-up. At the 6-month follow-up, they reported a significantly better color and overall appearance at the treated side. At the 12-month follow-up, the scores given were not significantly different between the treated and the control sides of the scar (Figures 1A-F, 2).

**Figure 3.**
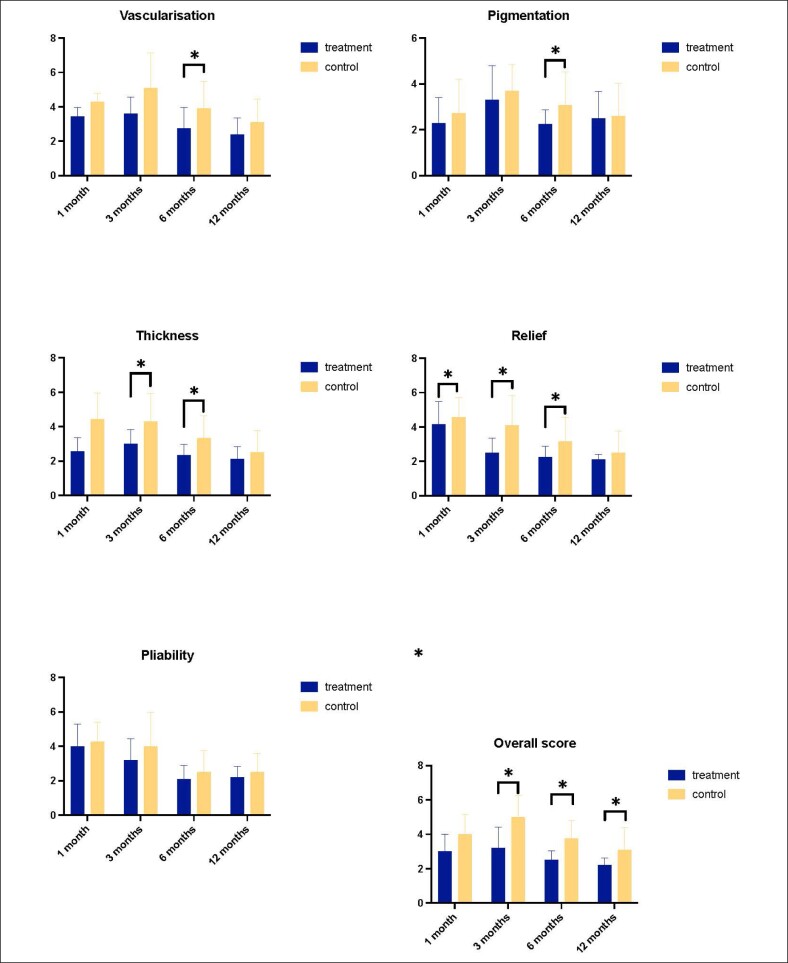
A graphical representation of observer-reported outcomes measured by using the Patient and Observer Scar Assessment Scale questionnaire (*P < .05).

Observers, blinded for the treatment, reported better texture (at 1, 3, and 6 months), better vascularization (at 3, 6, and 12 months), pigmentation (at 6 months), and thickness (at 3 and 6 months) on the POSAS questionnaire at the treated side of the scars. The overall appearance score was significantly better at 3, 6, and 12 months (Figure 3).

Two patients suffered from a small central wound dehiscence and received povidone–iodine wound dressings. No further adverse events were reported. There was no difference in the occurrence of adverse events between the 2 sides.

#### Improved Early Scar Maturation After Nanofat Infiltration Was Evident on Spectrophotometry by Significantly Lower Erythema Index Scores

**Figure 4.**
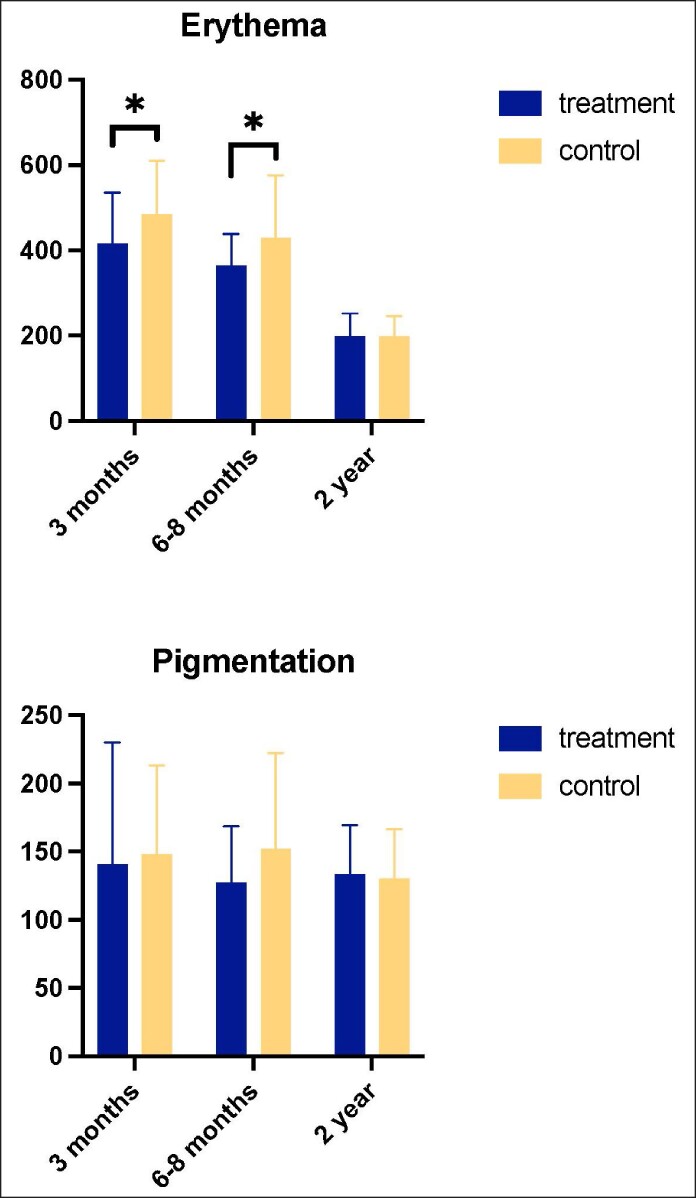
A graphical representation of erythema and pigmentation index scores measured by using the Mexameter (Courage + Khazaka electronic GmbH, Köln, Germany; *P < .05).

A paired 2-tailed analysis of the erythema index measured by using the Mexameter revealed that the abdominal scar was significantly less erythematous at the treated side at both 3-month (*P* < .05) and 6-month (*P* < .05) follow-up visits. This difference in hemoglobin content between the treated and control sides was no longer significant at 1-year follow-up visits (*P* = .99). The pigmentation index did not differ significantly between the treated and control sides at either the 3-month (*P* = .81), 6-month (.12), or 1-year follow-up visits (*P* = .41; Table 2, Figure 4).

**Table 2.** Erythema and Pigmentation Index Scores of the Treated and Control Sides of the Scar Measured by the Mexameter (Courage + Khazaka Electronic GmbH, Köln, Germany; *P < .05)

Age	
18-30	0
31-40	6
41-60	5
>50	2
Mean	43.9
Range	31-61
BMI	
18-25	8
25-30	5
Mean	25.1
Range	22.3-29.9
Sex	
Male	0
Female	13
Smoking	
Yes	0
No	13
Diabetes	
Yes	0
No	13

#### Histological Assessment Showed No Significant Structural Differences Between the Scar Tissue on the Treatment and Control Side

A total of 10 patients underwent tissue biopsy analysis for the evaluation of scar maturation at the 8-month follow-up visit. There was no significant difference in epidermal and dermal thickness or elastic fiber content between the treatment and control samples. Collagen staining and electron scan microscopy results show no statistical difference in 3-dimensional organization and collagen disposition between the treated and control sides (Supplemental Figures 1-6).

## DISCUSSION

Preclinical studies have mainly investigated the impact of SVF- and adipose-derived stem cell–based therapies on existing scars, in both the early and late remodeling phases and wound healing. Faster scar maturation was attributed to a decrease in myofibroblast and macrophage content, along with the induction of anti-inflammatory mediators such as transforming growth factor beta-β3 and remodeling mediators such as Matrix Metallo Proteinase-1 which decreases inflammation in the immature scar.^14,18,21^ Despite these promising in vitro properties, the translation to clinical studies has thus far provided limited evidence supporting therapeutic applications.^17,22^ Clinical studies investigating the effect on existing scars report improvement in color, stiffness, thickness, pliability, and pigmentation as measured by using the POSAS,^23-26^ whereas studies investigating the effects on wound healing report accelerated healing rates.^4,27,28^ Even when implemented in a topical cream, nanofat induced faster healing after laser resurfacing in a preliminary report by Cohen et al.^29^ Unfortunately, very few studies incorporated a within-patient control for comparison, which is the only study design that eliminates the numerous confounding factors that influence the process of wound healing and scar formation.

This pilot study evaluated the effects of a single intradermal nanofat administration in the fresh surgical wound on early and late scar formation. To our knowledge, it is the first preliminary report of an objective outcome measurement by means of spectrophotometry and histological properties of scar maturation after a single infiltration of mechanically isolated SVF in the surgical wound. Our findings suggest that SVF might be advantageous to shorten the inflammatory phase in early scar formation, but it showed no significant effect in the final remodeling phase or final scar outcome. Because none of our patients suffered from pathological scar formation, the possible effect of diminished inflammation in scar tissue in the prevention of hypertrophy and contracture was not evident in our study population. Our histological findings did not show significant differences between the treated and control sides, even when the treated side of the scar showed significantly lower erythema scores on spectrophotometry and lower vascularity scores on the POSAS at the 6-month follow-up. In a similar study, van Dongen et al infiltrated surgical scars after breast reduction surgery with mechanically isolated SVF. They also concluded that there was no evident difference in collagen distribution or alignment between the treatment and the control group at 6- and 12-month follow-ups. Similar to our results, they reported better POSAS scores at the treated side of the scar at the 6-month follow-up, but this advantage was no longer evident at the 12-month follow-up.^30^ Zivec et al did find an improved vascular density, elastic fiber content, and collagen maturity epidermal thickness and rete ridges on tissue biopsy after intraoperative infiltration of enzymatically isolated SVF compared with a non-treated side of the surgical scar at 10 months postoperatively, but they did not report any clinically evident benefit of their treatment protocol.^31^ The advantage on early scar maturation can be attributed to immunomodulatory properties of SVF, but the clinical benefit in the prevention and treatment of pathological scarring is not yet evident. Whether the reduced inflammation improves wound healing and, therefore, scar formation or tempers the inflammation in the immature scar is not yet clear. Wang et al found diminished fibro-inflammatory parameters such as macrophage and myofibroblast content after SVF infiltration in existing hypertrophic scars. They found that SVF gel, in which the ECM is preserved, induced more scar maturation than SVF cells alone. The most significant effects were seen at the 4-month follow-up.^18^ So far, there are no clinical studies at hand that investigate what the ideal timing of administration is, because SVF has been shown to be beneficial in the early wound healing phase as in the immature scar. This also raises the question whether multiple injections in the maturing scar would show a cumulative effect. In the same regard, there is still an unanswered question on the amount of SVF cells that should be administered in a clinical setting.^32-34^ In vitro and preclinical studies showed that a higher amount of SVF cells induce more angiogenesis, whereas other studies found increased fibrosis of fat grafts enriched with a high dose of SVF.^32,35^ The SVF cell yield per unit of adipose tissue is very variable between patients with age and BMI being the most discussed influencing factors.^36-38^ Also, cell yields are variable between different devices that mechanically isolate SVF, because the entrapment of fibrous remnants in filtering devices significantly reduces cell counts.^39,40^ The clinical repercussion of this variability between patients and devices is visible in the unpredictable clinical response of scars and wounds on SVF therapies. Therefore, larger clinical trials are needed that use comparable outcome measurement tools to evaluate the clinical impact of different cellular dosages. The applicability of this knowledge would still be challenging because a quantitative analysis of the cellular component before administration entails an important logistical bourdon that might jeopardize the bedside applicability of mechanically isolated SVF. There is also a growing consensus on the advantage of preservation of the extracellular matrix in which the SVF cells reside. The preservation of the inductive environment of the ECM and its growth factors and cytokines have shown superior advantages as opposed to single-cell suspension of SVF in preclinical studies investigating wound healing and hypertrophic scar therapies.^21,27,41-44^

This study is limited by the fact that it is a small study sample representing preliminary data. Because our study group did not contain any pathological scars, the clinical benefit of enhanced early scar maturation in the prevention of pathological scars should be investigated in larger randomized, controlled clinical trials. A preclinical cellular analysis of samples might help to clarify whether difference in dose accounts for the variability in clinical response to SVF treatments.

## CONCLUSIONS

The therapeutical ability to induce wound healing with minimal or no fibrosis would lead to improvements in the field of wound management and surgery. These preliminary clinical results suggest that the intradermal application of nanofat in the surgical wound improves early scar maturation, but the treatment did not result in better quality of the mature scar.

## Supplementary Material

ojae072_Supplementary_Data
