# Supplementary figures and images for: Improvement in Early Scar Maturation by Nanofat Infiltration: Histological and Spectrophotometric Preliminary Results From a Split Scar–Controlled, Randomized, Double-Blinded Clinical Trial
Source: Aesthet Surg J Open Forum. 2024 Aug 30;6:ojae072. doi: 10.1093/asjof/ojae072 (PMC11446608; doi:10.1093/asjof/ojae072)

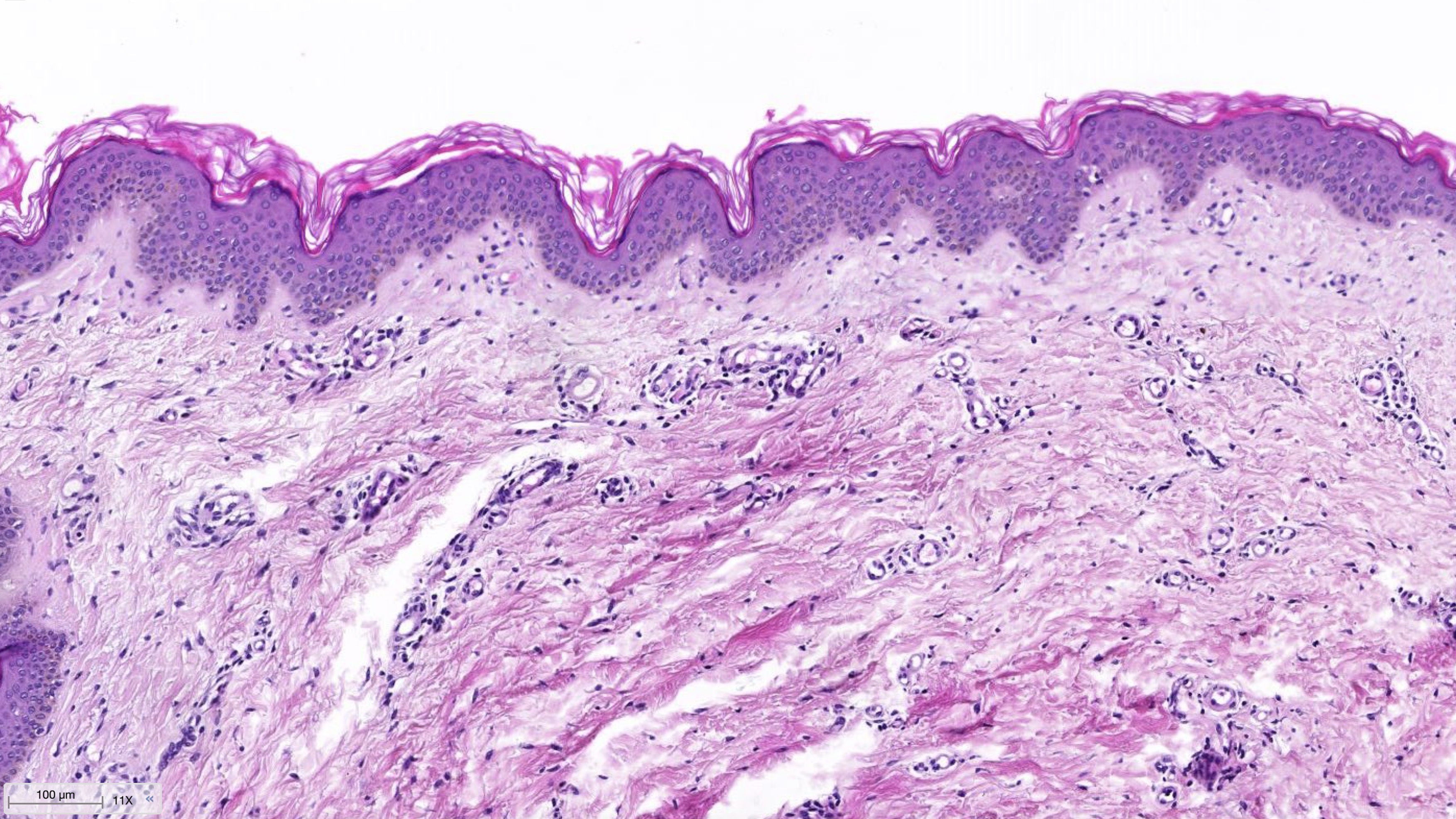

Supplement: ojae072_Supplementary_Data [file ojae072_supplementary_data.zip › SDC, Figure 1.jpeg]

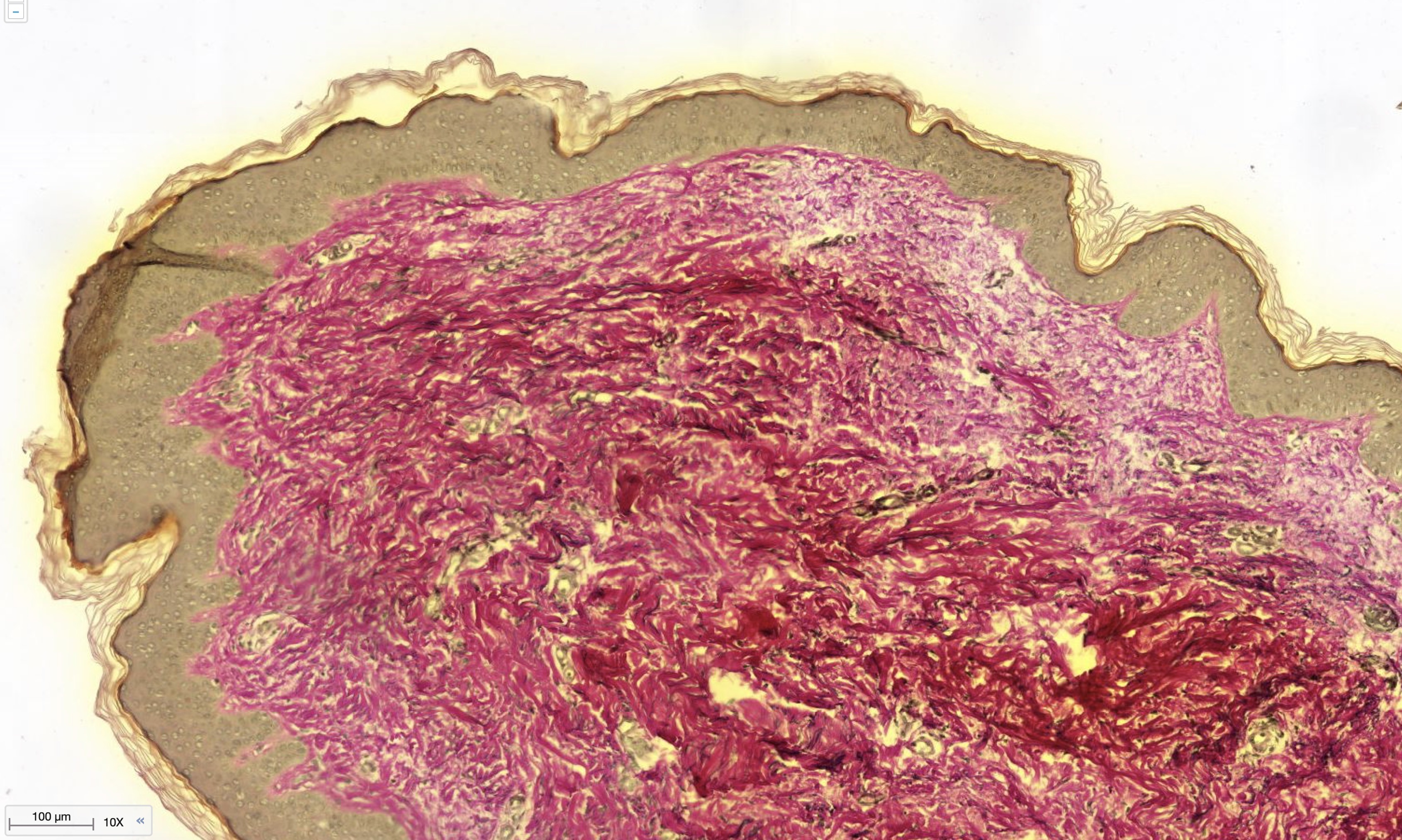

Supplement: ojae072_Supplementary_Data [file ojae072_supplementary_data.zip › SDC, Figure 2.jpeg]

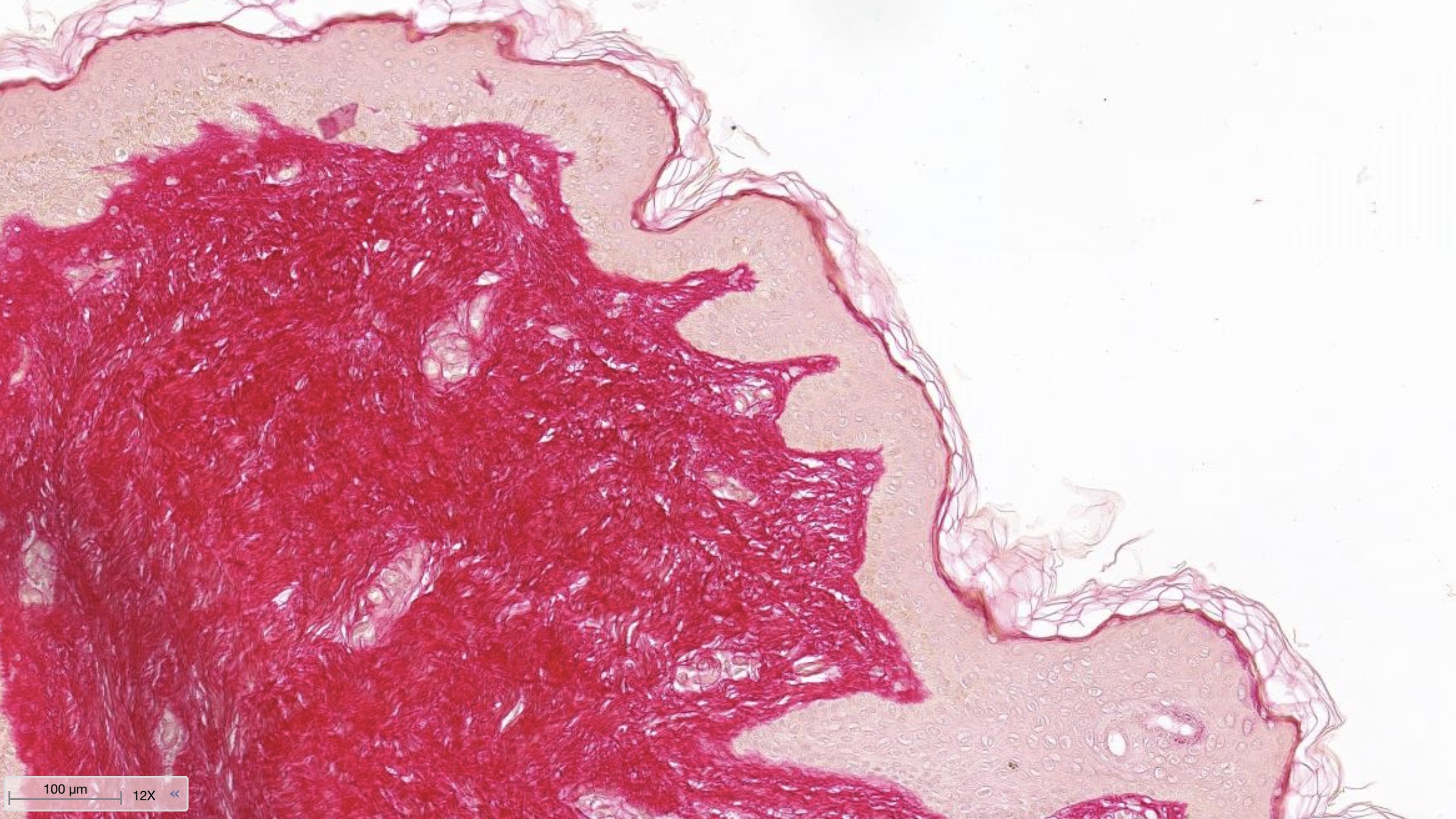

Supplement: ojae072_Supplementary_Data [file ojae072_supplementary_data.zip › SDC, Figure 3.jpeg]

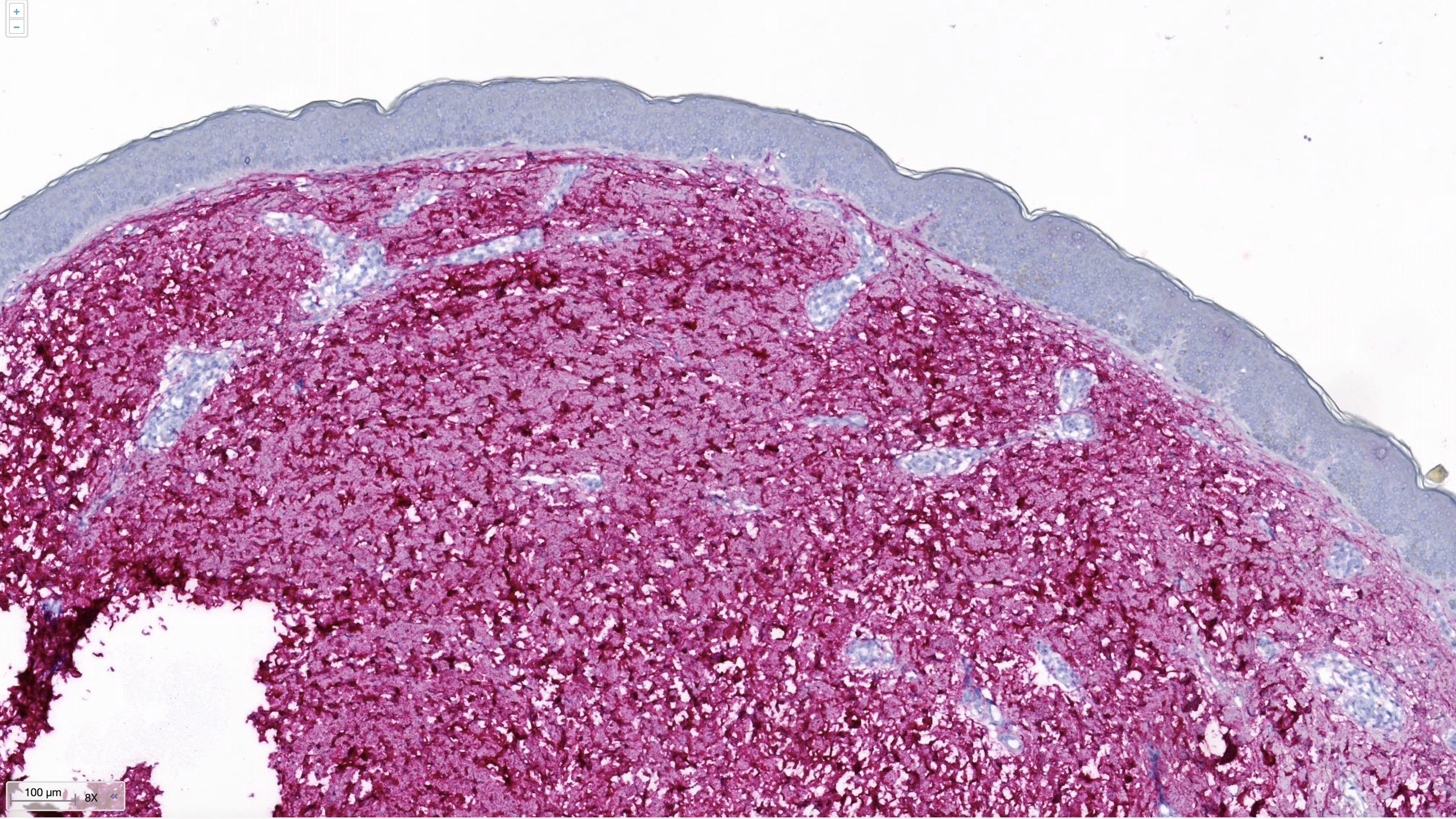

Supplement: ojae072_Supplementary_Data [file ojae072_supplementary_data.zip › SDC, Figure 4.jpeg]

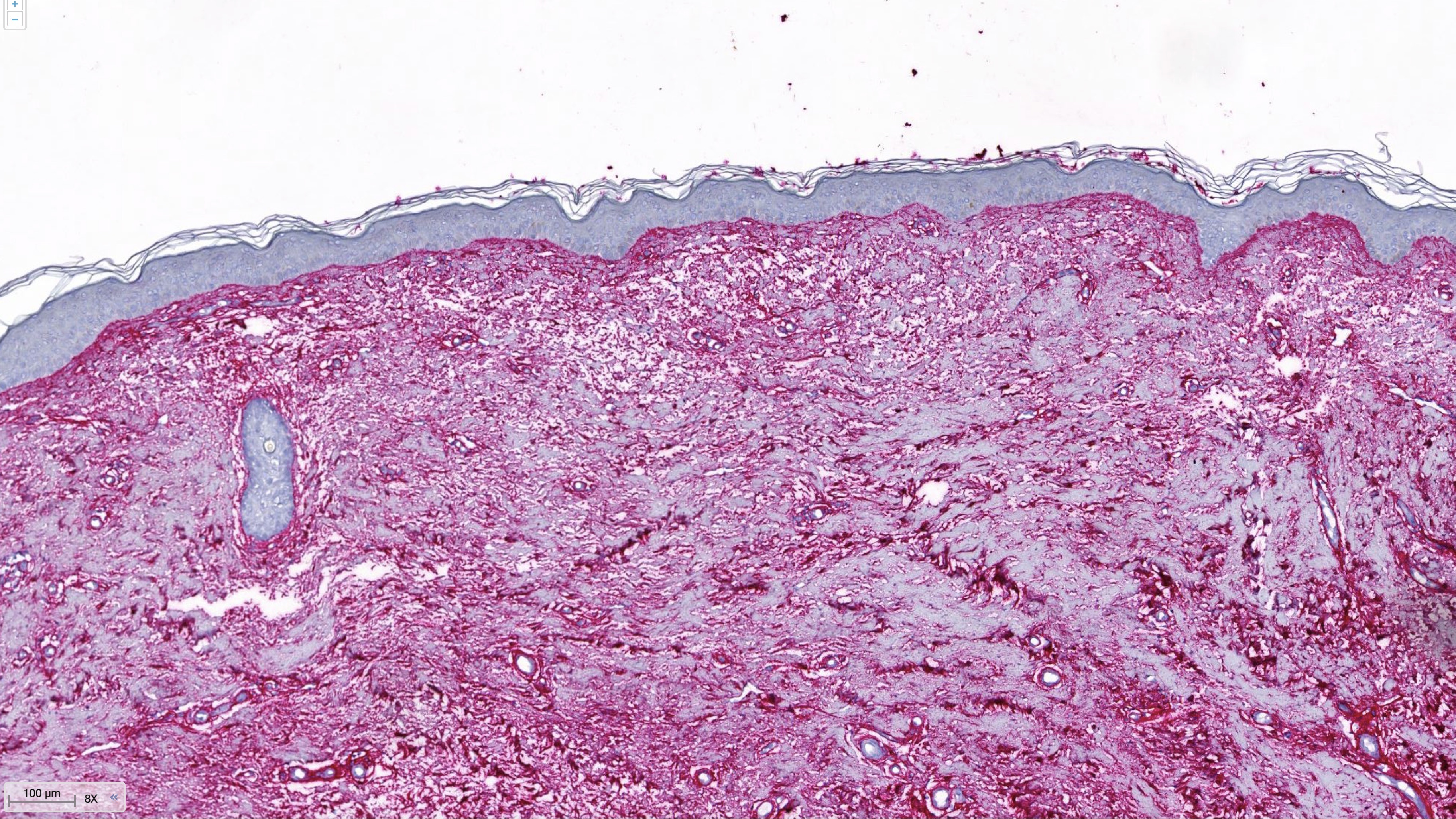

Supplement: ojae072_Supplementary_Data [file ojae072_supplementary_data.zip › SDC, FIgure 5.jpeg]

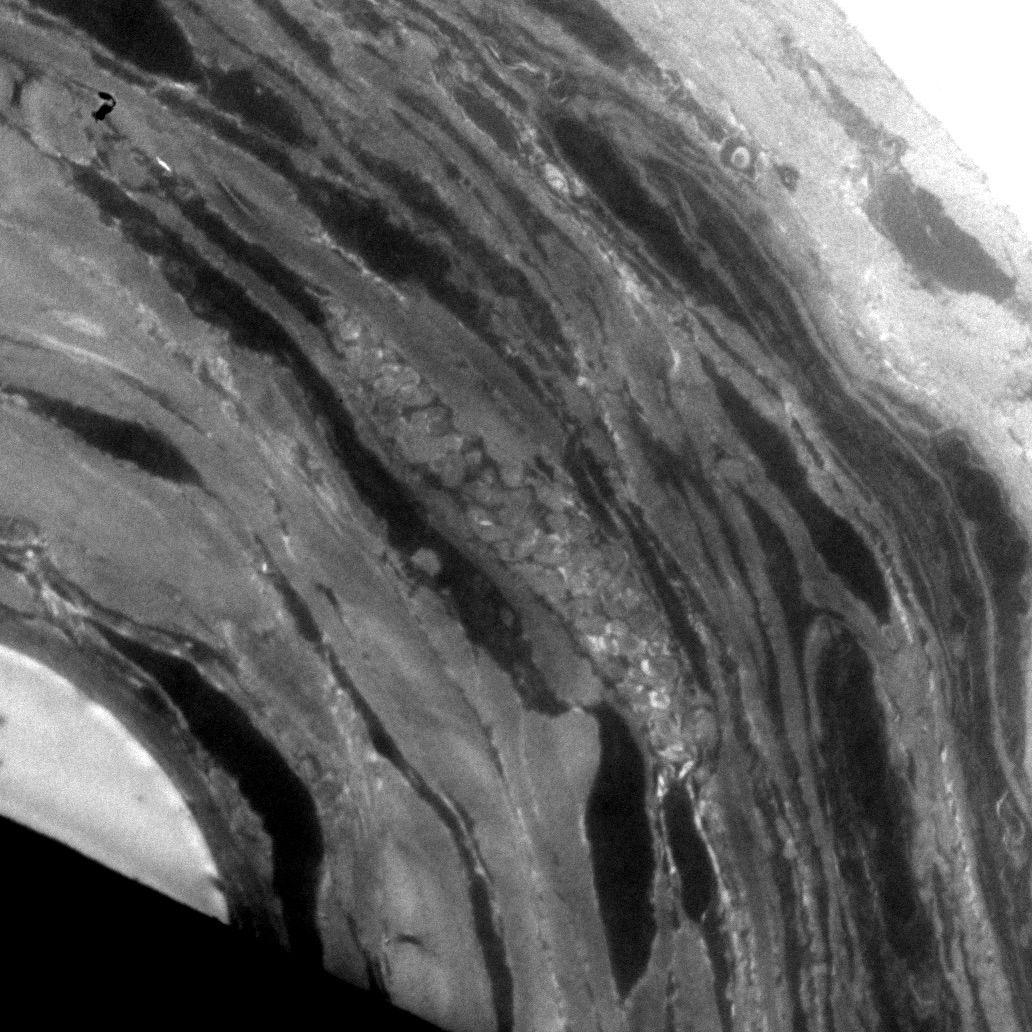

Supplement: ojae072_Supplementary_Data [file ojae072_supplementary_data.zip › SDC, figure 6A.tif]

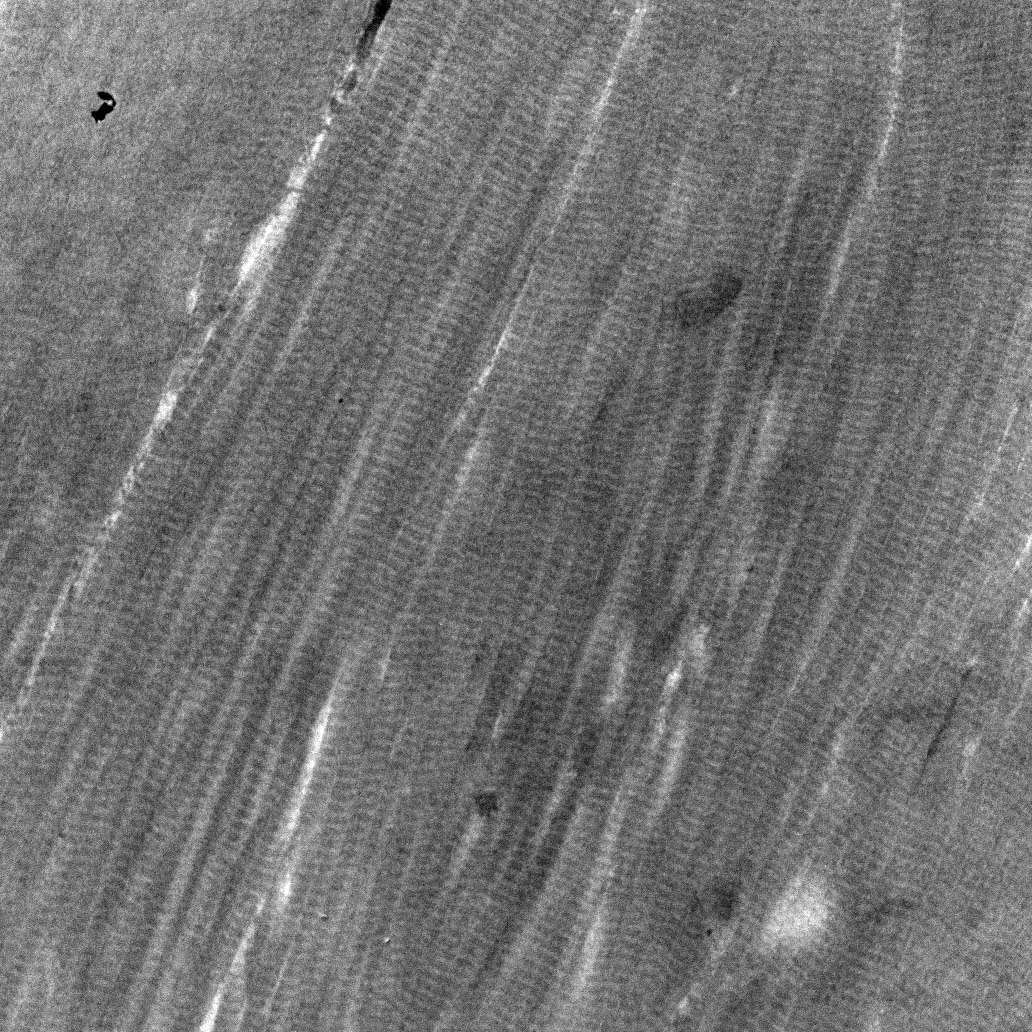

Supplement: ojae072_Supplementary_Data [file ojae072_supplementary_data.zip › SDC, Figure 6B.TIF]
